# Cervical Epidural Spinal Analgesia for Acute Management of Severe Unilateral Forelimb Lameness: Case Report

**DOI:** 10.3389/fvets.2021.749713

**Published:** 2021-11-04

**Authors:** Amanda R. Watkins, Klaus Hopster, David Levine, Samuel D. Hurcombe

**Affiliations:** Department of Clinical Sciences, New Bolton Center, University of Pennsylvania, Kennett Square, PA, United States

**Keywords:** epidural, morphine, analgesia, equine, opioid, supporting limb laminitis

## Abstract

A 20-year-old Quarter Horse gelding was presented with severe right forelimb lameness (5/5 AAEP Lameness Scale) due to a tear of the superficial digital flexor muscle which was diagnosed *via* palpation of swelling and ultrasonography revealing major muscle fiber disruption and hematoma formation. When traditional systemic therapy (non-Steroidal anti-inflammatories) did not restore clinically acceptable comfort and the risk of supporting limb laminitis became a reasonable concern, a cervical epidural catheter was placed between the first and second cervical vertebrae in the standing, sedated patient using ultrasound guidance. The gelding was treated with epidural morphine (0.1 mg/kg every 24 h then decreased to 0.05 mg/kg every 12 h) and was pain-scored serially following treatment. Spinal analgesia was provided for 3 days. Pain scores significantly decreased following each treatment with morphine, and the gelding was successfully managed through the acutely painful period without any adverse effects associated with the C1-C2 epidural catheter placement technique, the epidural morphine, or contralateral limb laminitis. At the 2-month follow-up, the gelding was walking sound with no complications seen at the catheter insertion site. In this case, spinal analgesia using epidural morphine administered *via* a cervical epidural catheter was an effective and technically achievable option for pain management associated with severe forelimb muscle injury in a horse.

## Introduction

Pain management for horses with severe unilateral forelimb lameness is challenging, and the consequences of ineffective analgesia provision can result in catastrophic consequences for the patient (1). Without appropriate pain control, horses are at risk for developing supporting limb laminitis due to unequal weight-bearing (1). The mortality rate associated with this condition is > 50% (1). The mainstays of treating forelimb pain include non-Steroidal anti-inflammatory drugs, which can cause ulceration of the stomach and the large colon in addition to renal crest necrosis (2), and opioids, which, when given systemically, have been shown to decrease colonic and jejunal motility and may cause behavioral changes including nervousness, pawing, and increased locomotion (3). Caudal epidural morphine administration has also been shown to temporarily increase GI transit time by 5 h in horses at a 0.2-mg/kg dose (4).

To reduce the risks associated with systemic opioid treatment and to increase their efficacy, these drugs can be used *via* epidural administration (5), either as a single injection or as longer-term therapy using an epidurally placed catheter (6). Placement of caudal epidural catheters for the administration of analgesic agents has proven to be safe and effective at providing longer-term hindlimb analgesia in horses (7, 8). However, the efficacy of caudal epidural catheter-administered therapeutics on forelimb lameness has not been proven, and higher injectate volumes are necessary to diffuse more cranial in the epidural space (9). Higher-volume epidural injections increase the risk of hypotension in other species (10, 11) which, in equine patients, can lead to dangerous ataxia and recumbency (9). In humans, single-dose cervical epidural steroid injections are used routinely in a clinical setting to treat neck or cervical spine pain (12), and cervical epidural catheters have been used experimentally (13). The use of cervical epidural catheters placed between the first and second cervical vertebrae using ultrasound guidance has recently been shown to be safe and technically straightforward in standing sedated horses (14). However, the clinical use of cervical epidural catheters has not been described in horses.

The objective of this report is to describe the use of a cervical epidural catheter in a clinical case for epidural morphine administration to manage severe pain as a result of a superficial digital flexor muscle tear in the forelimb of a horse.

## Case Presentation

A 20-year-old Quarter Horse gelding weighing 510 kg was referred to the University of Pennsylvania's New Bolton Center Large Animal Hospital with a history of acute, severe, non-Weight-bearing lameness of the right front limb. His pain level was severe enough to be mistaken for colic or an acute neurologic event. A physical exam revealed that the gelding was tachycardic at 80 beats per min. He was tachypneic at 44 breathes per min. His rectal temperature was within normal limits at 100.7° Fahrenheit. Colic and neurologic exams revealed no abnormalities in these body systems. Muscle fasciculations of the right triceps brachii muscle were present, and the caudal antebrachium was diffusely firm, with edema present medially from the olecranon to the accessory carpal bone (ACB). Radiographs of the cubital joint, radius, and radiocarpal joint were unremarkable. An ultrasonographic evaluation of the antebrachium revealed increased echogenicity of the superficial digital flexor (SDF) muscle from the musculotendinous junction proximally. There was loss of the normal architecture of the musculotendinous junction of the SDF. The disruption in the tendon fibers was most severe at 23 cm proximal to the ACB where 80% of the SDF muscle fibers were disrupted with loss of regularity and longitudinal orientation. The percentage of damaged fibers decreased but remained abnormal distally to the level of the ACB. The flexor carpi ulnaris muscle had interrupted muscle fibers in approximately 40% of the cross-Sectional area with the severity distributed similarly to the SDF muscle. In addition to fiber pattern disruption, many areas of mixed echogenicity and fluid pocketing were seen, consistent with hemorrhage, in both the SDF muscle and the flexor carpi ulnaris. A diagnosis of traumatic SDF and flexor carpi ulnaris muscle tears was made. The gelding was treated with phenylbutazone (4.4 mg/kg IV q12 h; VetOne, Boise, ID, USA), and a compression bandage was placed. The lameness remained unchanged following three doses of 4.4 mg/kg phenylbutazone IV every 12 h. Based on recorded findings in the medical record, 36 h after presentation, the gelding was spending more than 50% of the time recumbent.

Due to the lack of adequate responsiveness to systemic treatment and the > 50% of time spent recumbent, the decision was made to provide spinal analgesia by epidural morphine treatment. The gelding was sedated with detomidine (0.02 mg/kg IV, Zoetis, Parsippany, NJ, USA) for placement of a cervical epidural catheter as previously described (14). A 20 × 20 cm area to the right of the mane, caudal to the right ear, centered over the first cervical (C1) and second cervical (C2) intervertebral space was clipped and aseptically prepared. Ultrasound was used to localize the space between C1 and C2 and visualize the spinal cord, subarachnoid space, and paraspinal tissues. The skin and musculature deep to the proposed insertion site was locally anesthetized using 5 ml 2% mepivacaine (Carbocaine®, Zoetis, Parsippany, NJ). The ultrasound probe (Philips Lumify, C5-2 broadband curved array 2–5-MHz probe) was covered in a sterile plastic shield containing ultrasound transmission gel. A single operator (SDH) then controlled both the ultrasound probe and the 17-gauge 6-inch curve-tipped spinal needle with stylet (Tuohy). Using ultrasound guidance, the Tuohy needle was advanced dorsal to the probe until just adjacent to the dura. The stylet was removed, and 0.9% saline was placed in the hub. Next, the needle was advanced until loss of resistance and a positive hanging drop were observed. The epidural catheter (FlexBlock™, 19-gauge, 60 cm, Arrow International Inc., Reading, PA, USA) was then advanced through the spinal needle by a second operator (KH). The Tuohy needle was then withdrawn, removing the stylet of the epidural catheter with it. The first placement attempt was unsuccessful. There was increased resistance to passage of the catheter, and contrast radiography revealed subdural placement. This catheter was removed, and the horse was re-sedated. The second attempt with a new catheter was successfully placed in the epidural space with 6–7 cm of the catheter passed through the Tuohy needle. Correct positioning of the catheter was confirmed based on the loss of resistance, the hanging drop technique, the ability to pass the catheter caudally, and contrast radiography. The epidural catheter was then secured with cyanoacrylate glue at the insertion site, and the external length was sutured to the skin and secured using adhesive dressings.

Starting 36 h after the gelding's admission to the hospital, his pain was scored using a composite measure pain scale that has been previously described (15). This scoring system accounts for weight bearing, head carriage, location within the stall, gross pain behavior, and response to interaction. In addition to epidural morphine, the gelding was maintained on phenylbutazone (2 mg/kg PO every 12 h; VetOne, Boise, ID) for 7 days then 1 mg/kg PO every 12 h for 7 days.

**Figure 1:**
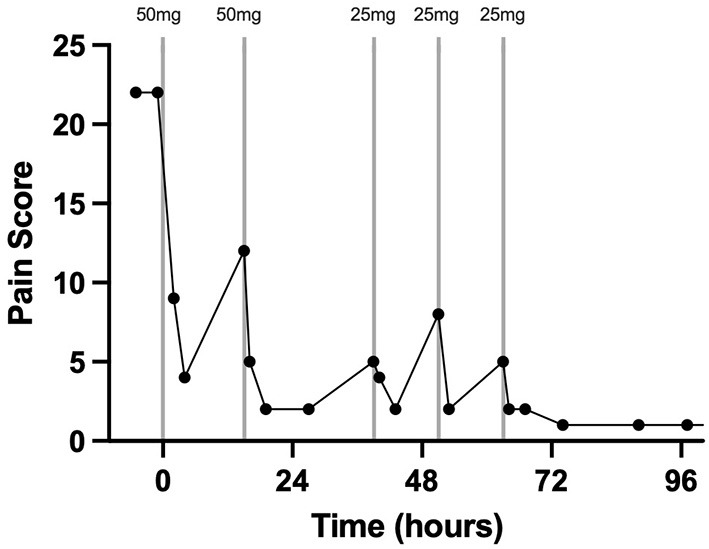
Pain scoring 2 h prior to, during, and after treatment with morphine *via* a C1-C2 epidural catheter in a horse. The vertical lines indicate the timing and dose of morphine administration. Each data point represents a pain score. Time zero is the time of C1-C2 catheter placement and first morphine dose.

**Figure 2:**
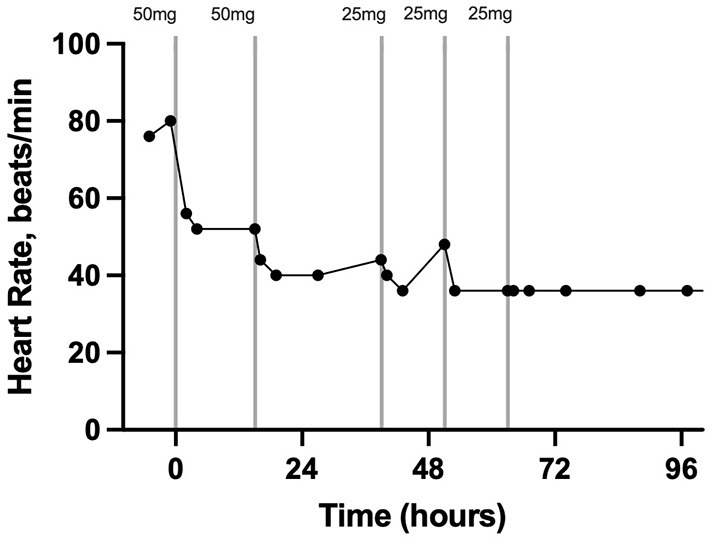
Heart rate in beats per minute 2 h prior to, during, and after treatment with morphine *via* a C1-C2 epidural catheter in a horse. The vertical lines indicate the timing and dose of morphine administration. Each data point represents a heart rate. Time zero is the time of C1-C2 catheter placement and first morphine dose.

Pain assessment and scoring were performed four times per day by a board-certified anesthesiologist (KH). The pain scores from just prior to epidural catheter placement until hospital discharge are depicted in detail in Figure 1. Prior to epidural catheter placement, the pain score was at the maximum allowed by the scale at 22. Thirty min following epidural catheter placement, 50 mg morphine sulfate (0.1 mg/kg BW; Henry Schein, Melville, NY, USA) diluted to a total volume of 5 ml with sterile saline was administered. Preservative-free morphine was not available at the time that this case was hospitalized. The morphine was administered with the gelding's head in an elevated position over a 30-min period to promote caudal movement of the epidural drug. The gelding's head remained elevated for 20–30 min following each morphine administration throughout his hospitalization. Twenty-four h after the second treatment, the morphine dose was reduced to 25 mg diluted to 5 ml with sterile saline over 20 min (0.05 mg/kg BW). This resulted in a pain score of 2/22 which increased to 5/22 by 12 h post-Administration; therefore, the same dose was repeated. The pain score remained acceptably low initially; however, by 12 h after the preceding morphine dose, a slight increase in pain was noted (score 5/22) and 25 mg diluted to 5 ml in sterile saline was administered a third time over 30 min. The gelding's time spent recumbent dropped to <10% 6 h after the first morphine dose. Throughout the remainder of the gelding's hospitalization, his pain score remained low (1–2/22) and no further morphine doses were administered. In response to epidural morphine, the heart rate decreased and remained within physiological limits for the remainder of his hospitalization (Figure 2).

The cervical epidural catheter was removed 48 h after the last morphine administration. There was no swelling or discharge from the catheter site while indwelling or following removal while in the hospital. At the 2-month telephone follow-up, the gelding was walking without lameness. The owner confirmed no heat, discharge, swelling, or pain at the insertion site of the catheter.

## Discussion

This case report describes the first use of a cervical epidural catheter to administer morphine to treat severe unilateral forelimb pain in a standing horse. The placement of these catheters has been described in research horses but never before in a clinical case (14). The graphical representation of the pain score and heart rate in addition to the percentage of time spent recumbent prior to and after treatment with epidural morphine indicates that this treatment effectively controlled significant forelimb pain. Additionally, the response in heart rate and pain score when the dose was reduced suggests strongly that the pain did not simply resolve coincidently with catheter placement but was controlled by the epidural morphine for intervals of 12 to 24 h when given at a 0.05 to 0.1 mg/kg dose.

Spontaneous rupture of the SDF tendon and/or muscle both at the proximal metacarpus and extending proximally into the carpal sheath and antebrachium has been reported in a wide age range of horses with a higher prevalence in older pleasure horses (16, 17). In the older population, with a mean age of 20 years, with lesions in the mid-metacarpus this diagnosis does not come with the grave prognosis of younger horses rupturing the SDFT during strenuous exercise more proximally within the carpal canal and antebrachium (16). All the horses in this case series returned to ridden activity, although mild carpal contracture was noted in all cases which was attributed to decreased weight bearing in the early healing period (16). Toppin *et al*. (18) report a case of incomplete superficial flexor muscle rupture with concurrent gastrocnemius muscle rupture in a similarly sized quarter horse that was treated with phenylbutazone, splinting, and management in a sling. This horse survived to discharged, but persistent discomfort and inability or unwillingness to evenly weight-bear resulted in a flexural deformity of the affected digit and contralateral limb laminitis. In summary, inadequate pain management can lead to complications such as flexural deformity and contralateral limb laminitis.

Muscular tears as primary causes of lameness are not well described in horses, but one retrospective study reported a positive outcome in 9/14 cases with negative outcomes being related to chronic lameness, initial degree of injury, and supporting limb laminitis (19). Interestingly, the report states that the initial degree of pain can be so severe in muscular tears that the lameness is mistaken for signs of colic, as occurred in this case when the gelding was referred for further treatment of colic and/or acute neurologic disease. The research on muscle tears and SDF tendon/muscle lesions highlights the overwhelming importance of treating the pain associated with these conditions expediently as the effects of asymmetric over-weighting of the contralateral limb can have severe consequences (16, 18). Supporting limb laminitis has been reported to occur in 12% of horses treated with half-limb, full-limb, or transfixation pin casts following a variety of surgical procedures including fracture fixation and pastern arthrodesis, with time spent in a cast being positively correlated with development of laminitis (20). In a paper evaluating fracture fixation and fetlock, pastern, and partial carpal arthrodesis using locking compression plates, 16% of horses experienced supporting limb laminitis in the postoperative period (21). The pathophysiology of supporting limb laminitis is not fully understood, but current literature suggests that reduced lamellar blood flow leading to ischemia and rotation or sinking of the distal phalanx is caused by a loss of normal cyclic loading of the digit (1) with changes occurring as quickly as 40 h after initiation of preferential weight bearing (22).

Placement of this catheter required two attempts to achieve correct anatomic positioning. The first catheter was determined to be outside the epidural space using contrast radiography. If there is any doubt in placement or in the hanging-drop technique, it is important to confirm placement using contrast radiography to ensure that the positioning is correct before administering therapeutics. Another positioning concern is retroflexion of the catheter tip cranially. If any resistance is encountered once the catheter has passed beyond the angled tip of the Tuohy needle, it is possible that the flexible tip of the catheter is retroflexed. This may result in resistance to injection despite correct positioning. The catheter can be placed either ipsilateral or contralateral to the affected limb with no difference in efficacy.

The use of morphine at the level of the spine makes intrinsic sense as the analgesic effect of morphine comes from activation of μ receptors at the interneuron level located within the dorsal horn of the spinal cord resulting in hyperpolarization and decreased pain impulse transmission (23). It is, however, not without complication; in a retrospective study examining 43 equine cases that were treated *via* caudal epidural catheters for a variety of reasons, there were technical issues associated with 44% of the catheters including dislodgement of the catheter, leakage, or obstruction of the catheter (8). Patient-Related complications, however, were much less common affecting only 6% of patients and being limited to inflammation or increased sensitivity around the catheter site (8). A common physiologic side effect of epidural drug administration in humans is hypotension due to sympathetic nervous system blockade and vasodilation (11). In horses, this might lead to sudden ataxia and recumbency which could be dangerous to the patient and to hospital personnel. Systemic morphine was not attempted in this case due to the shorter dosing interval leading to a higher total dose administered and because in this hospital's population it frequently is inadequate to provide analgesia to acute lameness cases. However, as systemic morphine was not administered, it cannot be said for certain that it would not have controlled the gelding's pain.

When forelimb pathology is treated *via* a caudal epidural catheter, a large volume of injectate is used to “push” the therapeutic agent cranial enough to affect the thoracic limbs. This large volume necessitates a longer administration time and therefore more time for the medication provider to be injured by a potentially hypotensive and/or ataxia patient. The cervical epidural catheter decreases the injectate volume to 5 ml which can be administered over a shorter period. The optimal injectate volume in these C1-C2 catheters to treat forelimb pain is yet to be determined and may be <5 ml.

Dural puncture is a possible adverse event associated with this epidural placement technique, as described by Hurcombe et al. (14). Horses can exhibit explosive reactions if dural puncture into the subarachnoid space is inadvertently performed, which can be dangerous for the patient, nearby personnel, and equipment (24). Post-Dural puncture headaches have been reported in humans, although not previously described in equine patients, which may cause neck soreness and back stiffness (25). The patient's head was maintained in an elevated position during administration of the epidural morphine over 30 min and for 30 min after administration to decrease the risk of seizures and/or sudden recumbency. It is not known what, if any, effects high concentrations of morphine have in the ventricles of the equine brain; therefore, to decrease the risk of unwanted side effects, these efforts to encourage caudal flow were taken.

In conclusion, the placement of an epidural catheter in the C1–2 space and provision of morphine were highly effective in providing spinal analgesia for forelimb pain in the horse of this report. This technique warrants further investigation in a larger number of clinical patients to assess both effectiveness of analgesia and potential adverse effects. Similar to caudal epidural catheterization, the cervical catheter can be placed in standing sedated horses, making this technique an attractive option to provide analgesia at the level of the spinal cord for the forelimbs and neck and with less reliance on systemic therapies. Further, lower total doses of morphine can be used due to the increased dosing interval compared to systemic morphine to potentially avoid decreased gastrointestinal motility while still providing adequate analgesia to allow to protect against the secondary effects of unequal weight bearing.

## Data Availability Statement

The original contributions presented in the study are included in the article/supplementary material, further inquiries can be directed to the corresponding authors.

## Author Contributions

All authors were involved in treating the case and involved in the writing and approval of the final draft.

## Conflict of Interest

The authors declare that the research was conducted in the absence of any commercial or financial relationships that could be construed as a potential conflict of interest.

## Publisher's Note

All claims expressed in this article are solely those of the authors and do not necessarily represent those of their affiliated organizations, or those of the publisher, the editors and the reviewers. Any product that may be evaluated in this article, or claim that may be made by its manufacturer, is not guaranteed or endorsed by the publisher.
